# Examining the Relationship Between Autism Traits and Sleep Duration as Predictors of Suicidality

**DOI:** 10.1007/s10803-020-04405-7

**Published:** 2020-02-21

**Authors:** K. D. Hochard, R. Pendrous, T. Mari, S. Flynn

**Affiliations:** 1 Centre for Contextual Behavioural Science, School of Psychology, University of Chester, Parkgate Road, Chester, CH1 4BJ UK; 2 Centre for Educational Development, Appraisal and Research, University of Warwick, Coventry, CV4 7AL UK

**Keywords:** Autism, Autism trait, Sleep duration, Suicide ideation

## Abstract

Short sleep duration is a known risk factor for suicidality in the general population, yet it is unclear how short sleep interacts with autism traits in predicting suicidality. In this cross-sectional online study, a general population sample (*N* = 650) completed measures assessing autism traits, suicidal ideation, and sleep duration. Moderated hierarchical regressions demonstrated that higher autism traits and shorter sleep were independent predictors of increased suicide ideation. However, sleep duration did not significantly moderate the autism trait to suicide ideation relationship. Future work should explore this relationship longitudinally using objective measures before considering intervention work to increase sleep duration in those with elevated autism traits.

## Introduction

### Suicidality in Autism Spectrum Conditions

Suicide is one of the largest causes of premature death in people with autism spectrum conditions (“autism” hereinafter), and the risk of death by suicide has been shown in a Swedish sample to be nine times greater for people with autism than in a matched sample of the general population (Hirvikoski et al. 2016). Although the exact risk for suicide ideation and attempts remain unclear (Zahid and Upthegrove 2017), reports estimate suicide ideation to occur in up to 72%, and suicide attempt between 7 and 47% of child, adolescent, and adult samples with autism spectrum conditions (Hedley and Uljarević 2018; Zahid and Upthegrove 2017). Adults with autism experience significantly more suicidal thoughts compared with adults in the general population (66% vs. 17%; Cassidy et al. 2014); and are more likely to attempt suicide more than once (Richards et al. 2019). Cassidy et al. (2018) have also indicated that people with autism report suicide risk scores significantly above the clinical cut-off compared to general population adults (72% vs. 33%). Further, Cassidy and colleagues reported that people with autism were more likely to report self-harm at some point in their life (65% vs. 29.8%). After statistically controlling for common demographic risk factors such as age and sex, the inclusion of autism diagnosis as a predictor accounted for an additional 4.5% variance in suicidality in the sample.

Autism traits are heterogenous, varying in severity within the general population (Landry and Chouinard 2016). That is, individuals may present with one or more behaviours or traits (such as impairment in social communication) relating to the broader autism phenotype (BAP; Piven, Palmer, Jacobi, Childress and Arndt 1997). Autism traits can be measured within the general population using the Autism Quotient (AQ; Baron-Cohen, Wheelwright, Skinner, Martin, and Clubley 2001). Those with an autism diagnosis have been found to score higher than the general population on this measure (Ruzich et al. 2015). BAP can be helpful in understanding autism. Research by Pelton and Cassidy (2017) using the AQ-50 has shown autism traits to be significantly associated with suicidal behaviours (*r* = 0.28) in a young adult nonclinical sample.

### Health and Psychological Impact of Short Sleep Duration

Sleep has been linked to most aspects of well-being, with disruptions in sleep duration being a predisposing factor for many health and psychological conditions. The National Sleep Foundation (NSF; Hirshkowitz et al. 2015) recommends 7 to 9 h for young adults and adults, and seven to eight hours of sleep for older adults. Sleep quantity, that is, the duration of sleep experience, should not be confused with sleep quality. The NSF (Ohayon et al. 2017) defines sleep quality as a subjective self-rated evaluation of the efficiency of the sleep period, which is frequently assessed as a composite of constituent objective elements, such as short sleep latency, reduced wakening after sleep onset, fewer awakenings, and longer sleep duration or total sleep time. Sleep duration is, therefore, a considerably narrower construct which has been associated with a range of health and psychological outcomes.

Meta-analytic and review evidence has demonstrated that short sleep duration increases the risk of negative health-related factors, including death (Cappuccio et al. 2010), with risk significantly increasing for those who reported sleeping six hours or less (Kripke et al. 2002), and obesity (Cappuccio et al. 2008). Similar risks of short sleep (< 5 h) have been associated with psychiatric disorders (e.g., mood disorders; Park et al. 2010), with significantly increased odds ratios for depression and anxiety disorders (John et al. 2005). It is possible that causality and direction of causality is potentially unclear due to likely comorbidity (e.g. Sun et al. 2018; Zhai et al. 2015). That said, experimental evidence has further demonstrated that prolonged sleep restriction (short sleep duration) follows a dose–response, with increased sleepiness and decreased cognitive performance (Van Dongen et al. 2003).

### Short Sleep Duration and Increased Risk of Suicidality

Systematic reviews have identified a range of sleep disturbances as empirical risk factors for increased suicidality (Bernert et al. 2015), including specific outcomes such as suicidal ideation, attempts, and suicide death (Pigeon et al. 2016). In their review, Pigeon and colleagues reported that cross-sectional studies have identified a relationship between shorter sleep duration and increased frequency or intensity of suicidal ideation, with one prospective cohort study (Gunnell et al. 2013) identifying that short sleep duration (< 6) was a key marker for increased suicide risk. Goodwin and Marusic (2008) demonstrated prolonged shorter sleep duration (how many hours usually slept in 24-h period) was associated with a 2.5 odds ratio (OR) increase in suicidal ideation, and a 3.0 OR increase in suicide attempt in a cross-sectional study of the general population (*N* = 8098). Lack of sleep has also been associated with self-harm risk in the general population. For example, one population-based study (Hysing et al. 2015) identified sleep problems (markedly lower sleep duration) as a pertinent warning sign for higher frequencies of self-harm. In particular, those who had never self-harmed had significantly more sleep (6.29 h) compared to those who had self-harmed (5.33 h). Here, the total amount of sleep of those who had self-harmed was lower than the NSF recommendations (Hirshkowitz et al. 2015). Further, a significant dose response was found between short sleep duration and frequency of self-harm; specifically, 37% of those who had self-harmed two or more times reported short sleep duration,, compared to those who reported only self-harming once (29%). Taken together, these findings suggest that longer sleep may be protective against self-harm and suicidal ideation (Hysing et al. 2015)**.** Longitudinal findings from ecological momentary assessment (repeated sampling in participants real time and natural environments; Shiffman et al. 2008) support these results, demonstrating that sleep duration is a significant predictor of next day suicidal ideation when using both objective (actigraphy, *β* =  − 0.09, *p* < 0.05) and subjective (a self-report sleep diary format, asking “time spent sleeping from intention to sleep to final wake time”, *β* =  − 0.08, *p* < 0.05) measures (Littlewood et al. 2018).

### Sleep Disturbance in Autism

The established associations between sleep problems and increased suicidality are especially relevant to people with autism, as the autism literature indicates people with autism have elevated levels of sleep problems compared to neurotypical controls (e.g. Baker and Richdale 2017; Richdale and Schreck 2009). Sleep disturbances are persistent in children with autism (Veatch et al. 2017), often leading to more severe symptoms of autism (Adams et al. 2014). The causes of sleep disturbances in this population are unknown, but they are likely multifactorial, a result of biological abnormalities, and intrinsic to the nature of autism (Mazzone et al. 2018). Parents have reported settling problems and insomnia in younger children, with estimates of 50% to 80% of children with autism experiencing sleep problems compared to 0% to 50% in matched controls (Richdale and Schreck 2009). Adams, Matson, and Jang (2014) found that the relationship between autism symptom severity and sleep problems was bidirectional in children with autism (*N* = 548), with children with more autism symptoms having greater difficulty sleeping. More specifically, sleep duration (*M* = 555 min) has been found to be negatively correlated with symptom severity in a sample of 2714 children with autism (Veatch et al. 2017). In this study, greater social impairments and more restricted and repetitive behaviours were more prominent in children with ≤ 420 min sleep per night compared to those having ≥ 660 min sleep. More recently, Salmela et al. (2019) showed that elevated autistic traits were independently predictive of a higher risk for short sleep duration after controlling for comorbid disorders in a sample of 157 adolescents. Moreover, when using both objective (actigraphy) and subjective (parent completed sleep diary) measures, Wiggs and Stores (2004) identified 63% of children with autism aged five to 16 years (*N* = 62) with a sleep problem.

Sleep difficulties in people with autism have been found to extend into adulthood (Matson et al. 2008) and are negatively associated with aspects of well-being, including; health-related quality of life (Delahaye et al. 2014) and quality of life generally (Deserno et al. 2019). This can lead to challenging behaviour (Goldman et al. 2011; Wiggs and Stores 1996), daytime functional impairments (Richdale et al. 2014), and a higher risk of meeting criteria for a circadian rhythm sleep–wake disorder compared to control age- and sex-matched adults (Baker and Richdale 2017). In a cross-sectional study using actigraphy, Baker and Richdale (2015) identified that adults with high functioning autism (*N* = 36) had greater sleep disturbances (especially lower sleep duration and longer sleep onset latency) compared to an age- and sex-matched control group (*N* = 36). Goldman et al. (2017) showed using longitudinal actigraphy that young adults and adolescents with autism had a significantly longer sleep onset latency than matched typically developing controls. Individuals with autism did not differ from controls on total sleep time (sleep duration), but the group did display larger standard deviations (53.0 min) compared to controls (26.2 min) demonstrating greater variability in sleep duration. However, research exploring sleep disturbances in people with autism tends to focus on younger to adolescent ages, and parent reports instead of self-reports. There is less of a focus on the sleep behaviours of adult with autism. No studies, to the authors’ knowledge, have investigated the potential interrelations between autism traits, sleep duration, and suicide ideation.

### The Present Study

Although the relationship between sleep duration and autism traits has not been explored in relation to suicidal ideation, it is reasonable to assume that there may be a similar association between short sleep duration and suicidality in people with elevated autism traits to the one seen in the general population (Littlewood et al. 2018; Hysing et al. 2015). Exploring the impact of sleep duration in this population has the potential to directly highlight directions for future research focusing on suicide prevention.

The present study aims to explore whether autism traits and sleep duration are predictive of suicidal ideation. Our study assesses this relationship beyond common demographic risk factors like age and sex (Cassidy et al. 2018). Finally, short sleep has negative health (e.g. Cappuccio et al. 2010), mental health (e.g. John et al. 2005) and cognitive functioning (e.g. Van Dongen et al. 2003) implications. We therefore also explore whether self-reported sleep duration buffers against the relationship between autism traits and suicide ideation. This is given that existing cross-sectional and longitudinal research has linked short sleep duration to increased suicidality (Hysing et al. 2015; Littlewood et al. 2018), and autism with sleep difficulties (Veatch et al. 2017). Based on the aforementioned literature, we hypothesize that, after controlling for age and sex:

1. Higher autism quotient scores will significantly predict increased suicide ideation.
2. Shorter sleep duration will significantly predict increased suicide ideation.
3. Sleep duration will moderate the relationship between autism traits and suicide ideation, with longer sleep duration reducing suicide ideation scores more so for those lower in autism traits.

## Method

### Participants

Prospective participants were recruited online using social media (e.g. Twitter, Reddit), and were required to be over 16 and fluent in English. No other inclusion or exclusion criteria were applied to this study, as such participants were not required to have a diagnosis of autism to take part. A total of 685 participants completed the survey. Participant data (*n* = 35) were excluded from the analysis whereby incomplete information prevented the computation of primary predictor (autism trait) and outcome (suicide ideation) variables. The final sample consisted of 650 participants (161 males) between the ages of 18–70 (*M* = 23.50, *SD* = 8.24). Of these, 143 (22%) participants had a diagnosis of autism, with the mean age at diagnosis being 21.66 years (*SD* = 12.38). Regarding comorbidities, of the 143 people who self-reported a diagnosis of autism, 38 had no other comorbidities, five did not provide an answer, 16 had ADHD/ADD, one had dyslexia, one had dyscalculia, two had dyspraxia, one had epilepsy, three had intellectual disabilities, three had OCD, three had tics, 39 had multiple comorbidities, and 31 reported “other” diagnoses (e.g. anxiety disorders, eating disorders, mood disorders). Further demographic information for our sample can be found in Table 1.

**Table 1:** Descriptive statistics and zero-order correlations for study variables (n = 650)

	*M*	*SD*	1	2	3	4	5	6	7
1. Age	23.50	8.24	–						
2. Sex (female)	489	–	.16^***^	–					
3. Autism traits	3.96	2.72	.24^***^	.32^***^	–				
4. AQ-short suggested diagnosis (Yes)	175	–	.25^***^	.33^***^	.86^***^	–			
5. Self-reported diagnosis (Yes)	143	–	.29^***^	.36^***^	.69^***^	.70^***^	–		
6. Sleep duration score (PSQI cat.)	.61	0.92	.09^*^	.09^*^	.22^***^	.16^***^	.09^*^	–	
7. Sleep duration (mins)	425	98.23	− .11^**^	− .07	− .15^***^	− .11^**^	− .06	− .837^***^	–
8. Suicide ideation	1.65	2.58	.06	.20^***^	.37^***^	.31^***^	.33^***^	.23^***^	− .17^**^

^***^p < .001, ^**^p < .01, ^*^ *p* < .05

### Materials

The 10-item version of the Autism Quotient questionnaire (AQ-10; Allison et al. 2012) was used to assess the degree to which participants have cognitive-behavioural traits related to autism. The AQ-10 was selected due to its brevity, and demonstrated specificity and sensitivity commensurate to the original AQ-50 (Booth et al. 2013). The use of this screening tool allowed for an accurate estimation of autism traits in the sample. Items are scored on a four-point Likert scale (strongly disagree to strongly agree). Higher scores on the AQ-10 indicate greater preponderance of autism traits. Further, established cut-offs (Booth et al. 2013) of (≥ 6) are suggestive of autism diagnosis. The internal reliability of the AQ-10 for the present sample was α = 0.78.

In addition to the AQ-10, a single dichotomous response (Yes/No) item “Do you have a diagnosis of Autism/Asperger's Syndrome?” was used to assess self-reported autism diagnosis.

Item four of the Pittsburgh Sleep Quality Index (PSQI; Buysse et al. 1989), “During the past month, how many hours of actual sleep did you get at night?”, was used to assess participant sleep duration. Sleep duration was reported in hours and minutes using a free-text response box. Responses were then converted into minutes to obtain a Sleep Duration (minutes) variable for our analyses. We also calculated responses on the PSQI four-point scale (0 = [> 7 h], 1 = [6–7 h], 2 = [5–6 h], 3 = [< 5 h]). This item was selected for its simplicity and high face validity. Further, it has previously demonstrated a mean high test–retest reliability of 0.80, with assessments taken between 2-days and 2-months (Backhaus et al. 2002).

Suicidal ideation was measured with the 4-item Depression Severity Index–Suicide Subscale (DSI–SS; Metalsky and Joiner 1997). Higher scores on the DSISS are indicative of an increased severity of suicidal symptoms over the past two weeks. Score ≥ 3 are indicative of clinical risk for suicide (Joiner et al. 2002). The scale has high face validity and good psychometric properties (Joiner et al. 2002; Metalsky and Joiner 1997), with a Cronbach’s alpha of α = 0.93.

To determine the presence of self-harm and suicide attempts in the sample, participants were asked two dichotomous (Yes/No) items: (i) “Have you ever hurt yourself on purpose in any way (for example, by taking an overdose of pills or by cutting yourself)?” and, subsequently, (ii) “On any of the occasions when you have hurt yourself on purpose have you ever seriously wanted to kill yourself?” These items were selected due to their high face validity (Mars et al. 2014).

### Procedure

This cross-sectional e-study was approved by the institutional ethics committee of the corresponding author. To allow for the broadest range of participants to take part, items selected for data collection were kept to a minimum to reduce participant burden. Participants were recruited by responding to an invitation to participate in an internet survey posted on social media platforms (e.g. Reddit). Having provided informed consent, participants completed a set of demographic questions (age, sex, diagnosis) followed by psychometric questionnaires. Participants received a written debrief on completion, including information on appropriate support services in line with the content of the study.

### Analysis Procedure

Our sample size calculation was informed by the reported effect sizes from Cassidy et al. (2014). We applied the smallest, most conservative reported estimate (Cohen’s *d* = 0.20, or *F*^*2*^= 0.02) to our calculations. To adequately power (1*-β* = 0.80, *α* = 0.05) a hierarchical linear regression (*ΔR*^*2*^) with three focal predictors (two main effects and one interaction term) from a model with a total of five predictors (controlling for age, sex) to detect a small effect, our calculation indicated that 550 participants would be required.

Statistical analyses were performed on SPSS v.25. Zero-order correlations were obtained for all interval data variables. To ensure the validity of our primary predictor variable, self-reported autism traits (AQ-10 scores), we dichotomized AQ-10 scores along suggested diagnosis threshold (≥ 6; Allison et al. 2012) to corroborate the validity of scores from an autism measure with self-reported diagnosis. A chi-squared test assessed the association between this suggested diagnosis (≥ 6/ < 6) variable and self-reported autism diagnosis (y/n). Further, exploratory chi-squared tests were performed to assess the association between suggested diagnosis and; history of self-harm, clinical risk (DSI–SS ≥ 3), and suicide attempt respectively. Odds ratios and 95% confidence intervals were obtained for all Chi-squared tests.

Inferential statistical testing for the interactions between autism traits and sleep duration (minutes) predicting suicide ideation was performed following the Aiken and West (1991) method. Autism traits and sleep duration were mean centered prior to computing the interaction term so as to ease the interpretation of the regression models.

Participant age and sex were entered at Step 1 of our regression analysis, allowing us to control for demographic variables. Autism traits scores and sleep duration were included at Step 2, allowing us to test hypotheses 1 and 2, namely that autism traits and sleep duration would be significant predictors of suicide ideation. The interaction product term between autism traits and sleep duration was entered at Step 3, allowing us to test hypothesis 3, that sleep duration will moderate the relationship between autism traits and suicide ideation. By entering our interaction term at Step 3, we are able to observe if the interaction provided a unique contribution in variance explained. The model was conducted with 5000 bootstrapped samples drawn from the data to obtain bias-corrected 95% confidence intervals (C.I.).

## Results

Means, standard deviations, and zero order correlations for study variables are reported in Table 1. Total sleep time in our sample was a median of 7.00 (1–14 h). Based on NSF recommendations (Hirshkowitz et al., 2015), *n* = 93 of our sample had less than the recommended total sleep time (≤ 5); *n* = 149 had between > 5 and < 7; *n* = 366 reached the recommended sleep time (≥ 7 and ≤ 9), and *n* = 42 had overslept (> 9 h). Of the 650 participants that provided complete responses, 614 participants opted to provide a response (Yes or No) for the self-harm measure, and 36 did not provide an answer. Of the 275 individuals reporting a lifetime history of self-harm, a total of 260 participants provided responses regarding suicide attempts.

A strong positive association was observed between AQ-Short suggested diagnosis and participant self-reported diagnosis of autism, (*χ*^*2*^[1] = 317.72, *p* < 0.001, φ = 0.70, OR = 49.77 [95% CI 28.90—85.69]). Moreover, a positive association between AQ-Short suggested diagnosis and self-harm (*χ *^*2*^[1] = 17.16, *p* < 0.001, φ = 0.17) was observed, with individuals meeting the threshold for diagnosis more than twice as likely to report lifetime self-harm (OR = 2.13 [95% CI 1.48–3.05]). We also observed a strong positive association between autism diagnosis and being at a clinical risk (DSI-SS score > 3) of suicide (*χ*^*2*^[1] = 60.09, *p* < 0.001, φ = 0.30, OR = 49.10 [95% CI 2.83–5.92]). However, the positive association we observed between having an AQ-Short suggested diagnosis and reporting a suicide attempt was not statistically significant, (*χ *^*2*^[1] = 1.38, *p* > 0.05, φ = 0.07, OR = 1.36 [95% CI 0.82–2.26]).

### Autism Traits and Sleep Duration Predicting Suicide Ideation

The regression coefficient at each step for models investigating the interaction between autism traits and sleep symptoms predicting current suicide ideation are displayed in Table 2.

**Table 2:** Moderated hierarchical linear regression of autism traits and sleep duration (Mins) predicting suicide ideation

Model	Variable	B	S.E. B	95% CI BCa for B		*β*
				Lower	Upper	
1. R^2^ = .04, *p* < .001	Constant	1.18	.30	.59	1.77	
	Age	.01	.01	− .02	.03	.03
	Sex	1.17	.23	.71	1.62	.20^***^
2. ΔR^2^ = .12, *p* < .001	Constant	1.91	.29	1.34	2.48	
	Autism traits	.87	.10	.68	1.07	.34^***^
	Sleep duration	− .31	.09	− .50	− .13	− .12^***^
3. ΔR^2^ = .003, *p* > .05	Constant	1.94	.29	1.36	2.51	
	Autism traits × sleep duration	.14	.09	− .04	.31	.06

^***^ *p* < .001, ^**^ *p* < .01

Our hierarchical moderated regression model (Table 2) explored the interaction between autism traits and sleep duration (minutes) on suicidal ideation, and predicted a total of 15.8% of variance (_adj_*R*^*2*^ = 0.16, *F*[5, 645] = 30.99, *p* < 0.001). Age and sex at Step 1 predicted 3.7% of variance (_adj_*R*^*2*^ = 0.04, *F*[2, 647] = 13.56, *p* < 0.001) in suicide ideation. The addition of the main effects of autism traits (*β* = 0.34, *p* < 0.001) and sleep duration (*β* =  − 0.12, *p* < 0.001) at Step 2 predicted an additional 11.9% variance (*ΔR*^*2*^ = 0.12, *F*[2, 645] = 46.50, *p* = 0.13) in suicidal ideation beyond participant age and sex. However, testing for moderation (*ΔR*^*2*^ = 0.00, *F*[1, 644] = 2.33, *p* = 0.13) by including the interaction effect at Step 3 revealed that the effects of autism traits on suicide ideation were not conditional on sleep duration (minutes) (*β* = 0.06, *p* = 0.127). The main effects of autism traits (*β* = 0.34, *p* < 0.001) and sleep duration (*β* =  − 0.13, *p* < 0.001) at Step 3 remained significant predictors.

## Discussion

The present study explored the relationship between autism traits, sleep duration, and suicide ideation in the general population. To our knowledge, this is the first study to explore short sleep duration as a potential risk correlate to suicide ideation in adults with autism traits. Hypothesis one, that autism traits would be associated with increased suicidality, was supported. Our analyses demonstrate that higher autism traits scores are predictive of increased suicidal ideation, beyond age and sex. These findings replicate those found in Cassidy et al. (2014) and Cassidy et al. (2018). Hypothesis two, that short sleep duration would predict increased suicide ideation, was also supported. Specifically, within our sample, shorter sleep duration predicted increased suicidal ideation, replicating findings previously seen in the wider literature (Littlewood et al. 2018; Pigeon et al. 2012). However, our third hypothesis, that sleep duration would moderate the relationship between autism traits and suicide ideation, was not supported. Specifically, the interaction between sleep duration and autism traits did not significantly predict suicide ideation, indicating that the risk of suicide ideation by individuals with high autism traits was not conditional on short sleep duration. The slight increase in the *β* coefficient of the main effects at Step 3 of our model is likely due to the additional error variance accounted for by the non-significant interaction effect.

Our results also indicated that shorter sleep duration (minutes) was associated with more autism traits (*r* =  − 0.17, *p* < 0.001) and having an autism diagnosis (*r* =  − 0.11, *p* < 0.01). Although the correlations are small, these results are in line with previous research about the relationships between autism and reductions in sleep time (Baker and Richdale 2015; Salmela et al. 2019; Veach et al. 2017), and day-time functioning impairments (Richdale et al. 2014). One education-based study (Loring et al. 2016) using sleep hygiene and cognitive behavioral techniques (CBT-I) with a sample of adolescents with autism and demonstrated significant improvement via actigraphy measurement. Meta-analyses (e.g. Van Straten et al. 2017.) do show CBT-based interventions to be effective in improving a range of sleep variables. Sleep interventions for adults with autism could therefore be beneficial, though the normative ranges of sleep duration for people with autism are unknown (Loring et al. 2018).

Exploratory Chi-square analyses demonstrated that there was a positive association between suggested autism diagnosis (AQ scores) and lifetime self-harm as well as suggested autism diagnosis and suicide clinical risk (based on DSISS cut-offs; Joiner et al. 2002), replicating findings in Cassidy et al. (2018). Further, Pelton and Cassidy (2017)’s findings that autism traits are significantly associated with suicidal behaviours are corroborated by our findings. However, our exploratory findings did not replicate Richards et al. (2019) in that the association between suggested autism diagnosis and lifetime suicide attempt was non-significant. It is possible that the exploratory Chi-square analyses between suggested autism diagnosis and suicide attempt were non-significant because of the smaller number of participants providing data regarding suicide attempts (*n* = 260). Only those who had previously engaged in self-harm were required to answer this question. Thus, analyses including our suicide attempt variable were likely underpowered. This reduction in our sample would lead to reduced statistical power for this analysis. Taken together, these results demonstrate that findings within the autism-suicide literature appear robust and replicable for suggested autism diagnosis, self-harm and suicide clinical risk. However, further attempts to replicate findings relating to suicide attempts should consider the large sample required to recruit sufficient numbers of those who have attempted suicide, due to its low base rate in the general population (Cohen 1986).

### Study Evaluation

There were limitations worthy of note in this study. First, all results are based on self-report, which are inherently subject to memory bias. In particular, self-reported sleep can be problematic due its retrospective nature which might lead to over or underestimations of sleep duration. Second, the measurement tools used to assess suicidality are not yet validated in people with autism (Cassidy et al. 2018). The current study used the DSI-SS (Metalsky and Joiner 1997), which has been validated in an Australian population presenting to practitioners aged between 15–24 (*N* = 2851), with a standard normative cut off of ≥ 3 that is indicative of clinical risk of suicide (Joiner et al. 2002). It is presently unknown if a cut-off of ≥ 3 on the DSI-SS is sufficiently sensitive and accurate in identifying clinical risk in a population of people with autism. Relatedly, sleep duration was assessed using a using single-item psychometric measure. Although such short measures could reduce burden on participants, they render us unable to account for measurement error and calculate reliability for sleep duration (Nunnally and Bernstein 1994). The PSQI (Buysse et al. 1989) only contains a single item relating to sleep duration (item 4, used within this study). This item is high in face validity, but our assessment of sleep duration using the PSQI item 4 suffers from an inability to establish an index of reliability.

Granting this may be of concern, single item measurements should not be dismissed out of hand. Measurement error can be increased by relying on single items, but measurement error is more prevalent for abstract than for concrete constructs (Cote and Buckley 1987). A construct may be suitably captured by a single-item measure if said single-item reflects a narrow-band, homogenous and unidimensional construct (cf. Clark and Watson 1995; Loevinger 1957), such as a concrete object like sleep duration. Few stimuli, other than item 4 (presently used), could legitimately evoke the requisite response from the participant, without being redundant. Given this, we argue that a single item sleep duration measure is justified.

Finally, the AQ-10 allowed for autism traits to be measured on a continuum and this was important when assessing the level of autism traits in relation to the severity of suicidal ideation, however, this may have decreased the power of moderation analyses (McClelland and Judd 1993). We are confident that our findings limit the likelihood of Type 2 errors to 20% as we obtained a sufficiently large sample to meet a priori sample size recommendations. As this is the first study addressing this specific research question, direct replications studies would strengthen the reliability of these findings. However, to achieve this sample size, our sampling method relied on social media recruitment. This likely precluded low functioning individuals from participating. Indeed, our demographics indicate that only three individuals with self-reported intellectual disabilities participated. As such, our findings may generalize to high functioning individuals with autism traits but should be interpreted very cautiously with regards to low functioning individuals.

### Future Directions

This study identified short sleep duration as a risk correlate for people irrespective of their autism traits levels. We strongly recommend that future studies use longitudinal designs, to allow for the assessment of sleep duration as a causal risk factor for suicidality in this sample, according to the typology outlined in Kraemer et al. (1997). It is possible that using ecological momentary assessment with objective measures such as actigraphy, as used in Littlewood et al. (2018), would give more accurate assessments of sleep duration, as well as other sleep variables (e.g. latency, efficiency, number of awakenings) beyond that of the self-report in the present sample. Further, actigraphy devices have continuous recording capabilities which would yield more data without increasing participant burden. The combined use of diary self-report and actigraphy would also provide multiple measures of sleep duration, thus providing parallel tests (Nunnally and Bernstein 1994) addressing some of the issues of reliability discussed previously. Future studies could also use the full PSQI or measures of specific sleep disorders associated with suicidality such as insomnia or nightmares (Pigeon et al. 2016), to assess other facets of sleep quality, beyond sleep duration. Moreover, developing and validating existing measures for an autism population, and direct replication studies are recommended.

Finally, it is probable that sleep disturbance may be characterised differently in those with or without intellectual disabilities, however this was beyond the scope of the present study. That said, recruiting those who may have comorbid intellectual disabilities may be difficult to do using psychometric methods. Future studies may wish to consider exploring how intellectual disabilities relate to suicidality and sleep disturbances, particularly using observational methods.

## Conclusion

This is the first study to assess short sleep duration, an established suicide risk factor (Littlewood et al. 2018), as a unique correlate of suicide ideation in a general population sample while accounting for autism traits. Our analyses demonstrated that short sleep duration was associated with suicide ideation, as were autism traits, beyond known demographic risk factors of autism (i.e. sex, age). However, this relationship was not moderated by (i.e. there was no interaction) sleep duration in this sample. Future research should assess the impact of sleep on suicidality in autism samples, using longitudinal designs with behavioural measures. This would clarify if interventions targeting sleep outcomes would yield salutary effects in reducing suicide risk for people high in autism traits. The association between autism traits and suicide ideation in the present study was not conditional upon levels of sleep duration. That said, short sleep duration has broad adverse health-related implications (e.g. Cappuccio et al., 2010), and can lead to cognitive functioning impairments (Van Dongen et al. 2003), in the general population. Interventions in general that improve sleep duration would benefit health and potentially reduce suicide ideation (Bishop et al. 2019), regardless of autism traits.
